# Distribution and classifications of *PKHD1* gene variants in a Turkish population using the next generation sequencing method

**DOI:** 10.55730/1300-0144.5892

**Published:** 2024-05-23

**Authors:** Yüksel GEZGİN, Berkay KIRNAZ, Rauf BAYLAROV, Afig BERDELİ

**Affiliations:** 1 Division of Molecular Medicine, Department of Pediatrics, Faculty of Medicine, Ege University, İzmir, Turkiye; 2 Department of Pediatrics, Faculty of Medicine, Ege University, İzmir, Turkiye; 3 Division of Pediatric Nephrology, Department of Pediatrics, Azerbaijan State Medical University, Baku, Azerbaijan; 4 Division of Pediatric Rheumatology, Department of Pediatrics, Faculty of Medicine, Ege University, İzmir, Turkiye

**Keywords:** Autosomal recessive polycystic kidney disease, *PKHD1* gene, next generation sequencing, missense variant, prenatal diagnosis

## Abstract

**Background/aim:**

Autosomal recessive polycystic kidney disease is an inherited kidney disease. This study aims to detect rare and common DNA variants of the *PKHD1* gene using next-generation sequencing (NGS) and to classify them in terms of being pathogenic according to The American College of Medical Genetics and Genomics.

**Materials and methods:**

NGS analysis was performed on the DNA of 304 patients who were referred to Ege University Molecular Medicine Laboratory with suspected polycystic kidney disease.

**Results:**

As a result, a total of 82 different DNA variants, 16 of which were novel, were detected. The breakdown of the variants found is as follows: 73 (89.02%) were missense variants, six (7.32%) nonsense variants, two (2.44%) frameshift deletions, and one (1.22%) nonframeshift deletion. According to The American College of Medical Genetics and Genomics classification of these variants, 26 were benign (Class 5), two were likely benign (Class 4), 36 were of uncertain significance (Class 3), and nine were likely pathogenic (Class 2), nine of which are pathogenic variants (Class 1). Heterozygosity was found in 39 (63.9%) patients, homozygosity in six (9.8%) patients, compound heterozygosity in 12 (19.7%) patients, and complex genotype in four (6.6%) patients in which variants in Class 1, Class 2 and Class 3 were determined according to ACMG classification. When the exon distributions of the DNA variants detected in the *PKHD1* gene were analyzed, the most common exons of the DNA variant are exon 32 (n = 9), exon 58 (n = 8), exon 67 (n = 6), exon 61 (n = 5), 30 exons (n = 4).

**Conclusion:**

This fast and economical molecular diagnostic approach will provide a reliable prenatal diagnostic option, enabling definitive disease diagnosis and the identification of carriers.

## 1. Introduction

Autosomal recessive polycystic kidney disease (ARPKD, OMIM:263200) is a hereditary polycystic kidney disease that occurs in childhood with a prevalence of 1:20,000 in live births [1,2]. ARPKD occurs earlier than another hereditary nephropathy, autosomal dominant polycystic kidney disease (ADPKD), and presents with a more severe clinical course. Most cases can be diagnosed late in the pregnancy or at birth. Approximately 30%–50% of newborns affected by this disease die shortly after birth from respiratory failure due to pulmonary hypoplasia and thoracic compression of their overgrown kidneys [3,4].

The *PKHD1* (fibrocystin/polyductin) gene on chromosome 6, which is responsible for ARPKD, was first identified in 2002 [5,6]. This gene region encodes a protein of 4074 amino acids called fibrocystin [5] which is found in fetal and adult kidney cells. It is also expressed, albeit at lower levels, in cells in the liver and pancreas. Like other cystoproteins (PKD1 and PKD2), fibrocystin is localized in the basal body and the primary cilia of kidney cells [3,4]. Fibrocystin is thought to function as a key regulator of cell proliferation, apoptosis, and polarization, as well as playing a role in cell-matrix and cell-cell interactions [7]. Although it is known that the DNA variants occurring in *PKHD1* prevent the function of the fibrocystic protein found in the primary cilia of the kidney [8], the molecular mechanism of cyst formation remains to be elucidated [3,4,9].

According to HGMD data [10], 800 variants of *PKHD1* have been reported. Establishing a genotype-phenotype correlation is extremely difficult since the *PKHD1* gene is large, and a wide variety of genetic variants can occur. However, extremely varied *PKHD1* DNA variants were detected by screening the DNA variants occurring in the *PKHD1* gene using the Next Generation Sequencing method. Such studies contribute to better understanding the genotype-phenotype correlations that exist with the disease [11].

In this study, DNA rare and common variants of the *PKHD1* gene in the Turkish population were identified using next generation sequencing. Pathogenic classification of these detected DNA variants was made using the ACGM guide and in silico approaches.

## 2. Materials and methods

### 2.1. Study population

Our study included 304 patients suspected by clinicians of having polycystic kidney disease who were referred to Ege University Children’s Hospital Molecular Medicine Laboratory. Ethical approval was provided by the Ethics committee of Ege University, İzmir, Türkiye (22-IT/19). This study was conducted in accordance with the Helsinki Ethical Standards.

### 2.2. Sample preparation and targeted next generation sequencing analyses

Genomic DNA was obtained from peripheral blood samples of patients using the PureLink Genomic DNA Mini Kit (Thermo Fisher, Waltham, MA, USA). DNA concentrations were first equalized for library preparation, and then DNA samples were amplified with predesigned primer pools using the Ion AmpliSeq Library Kit 2.0 (Thermo Fisher). Amplicons were barcoded with the Ion Express Barcode X kit and purified using AMPure XP reagent, and ethanol. The amplicons were quantified with QUBIT 2.0 (Invitrogen, Carlsbad, CA, USA) to equalize the concentrations. Template preparation was performed using the Ion OneTouch 2 device (Ion Torrent, Guilford, CT, USA) according to the Ion PGM Template OT2 400 Kit (Ion Torrent) protocol. The amplicons were loaded on the chip (Ion 318 Chip v2 BC) and sequencing based on semiconductor sequencing technology with the PGM Hi-Q (Ion Torrent) kit on the Ion PGM System (Ion Torrent). The data were taken from the Ion Reporter program and evaluated. GRCh37/hg19 was used as the reference genome. The American College of Medical Genetics and Genomics (ACGM) classification [12] was applied according to Franklin Genoox (https://franklin.genoox.com/clinical-db/home).

### 2.3. Predicting the impact of DNA variants

The pathogenic effects of missense variants were determined using bioinformatic tools such as Polyphen2 and SIFT [13,14].

## 3. Results

This study detected a total of 82 different DNA variants in 304 patients, 16 of which were novel (Table 1). The domain distributions of these DNA variants in the *PKHD1* protein are provided in Figure. Of these variants, 73 (89.02%) are missense variants, six (7.32%) are nonsense variants (p. Asn711Ter, p.Arg3961Ter, p.Arg494Ter, p.Arg592Ter, p.Ser2639Ter, p.Arg3107Ter), two (2.44%) are frameshift deletions (p.Gln256ArgfsX63 and p.Leu2764fsX67), and one (1.22%) is nonframeshift deletion (p.Ser1929_Arg1930del) (Table 1).

According to ACMG classification of these variants: 26 are benign (Class 5), two are likely benign (Class 4), 36 are of uncertain significance (Class 3), nine are likely pathogenic (Class 2), and nine are pathogenic variants (Class 1) (Table 1). Accordingly, as a result of the ACMG classification, a total of 61 different variants in Class 1, Class 2, and Class 3 were identified. All these variants and their genotypic distributions are presented in Table 2.

The genotype distribution of *PKHD1* DNA variants was then determined, including Classes 1, 2, and 3 in the analysis. The distribution was as follows: 39 patients (63.9%) were heterozygous, six patients (9.8%) were homozygous, 12 patients (19.7%) were compound heterozygous, and four patients (6.6%) had a complex genotype, categorized into Class 1, Class 2, and Class 3 (Table 2).

When the exon distributions of the DNA variants detected in the *PKHD1* gene are examined in the study, the distributions of the exons with the most common DNA variant are as follows: exon 32 (n = 9), exon 58 (n = 8), exon 67 (n = 6), exon 61 (n = 5), exon 30 (n = 4) (Tables 1 and 3).

## 4. Discussion

Gene-based studies related to autosomal recessive polycystic kidney disease (ARPKD) help identify cases with mild clinical course and atypical symptoms by detecting common DNA variants that cause this disease and rare mutations [15–17].

Screening studies of this type also provide helpful information for the genotype-phenotype correlation of the disease [9,15,16,18–20]. While many DNA variants of the *PKHD1* gene have been described (Table 4), *PKHD1* mutations that are common in specific populations may differ [15–17,21–34].

*PKHD1* mutations may vary depending on the geographic origin of the patient. They are collected in specific exons [28], possibly related to environmental and genetic modifiers [16,35,36]. Detecting these mutations, creating a mutation profile of the population specific and/or common *PKHD1* gene, and identifying the exons with relevant mutations [23], significantly increases the efficiency of genetic testing for ARPKD [23,28]. This study defined exons in which variants of the *PKHD1* gene were seen in the Turkish population cluster/aggregate (Table 3). In a study by Sharp et al., it was stated that most mutations were detected in exons 32, 59, and 65 [23]. In another study, 4 missense mutations were detected as a result of analysis performed on individuals with suspected disease (c.107C > T, p.(Thr36Met); c.406A > G, p.(Thr136Ala); c.4870C > T, p.(Arg1624Trp) and c.9370C > T, p.(His3124Tyr)), with these mutations being identified in exons 3, 6, 32, and 58, respectively [37]. In this study, the largest number of mutations were observed in exon 32 (n = 9), exon 58 (n = 8), exon 67 (n = 6), exon 61 (n = 5), and exon 30 (n=4).

Studies show that different *PKHD1* DNA variants are dominant in different ethnic groups. For example, in a study on the Finnish population, the incidence of R496X and V3471G mutations of the *PKDH1* gene was reported to be 60% [36]. In another study, the c.9689delA mutation was observed in 34% of Hispanics [21,28]. Neither of these mutations was found in this study.

The T36M mutation in the *PKHD1* gene is the most commonly known mutation typically associated with a severe phenotype [15,18,37,38]. This rate varies across studies. According to Goggolidou and Richards, this mutation accounts for approximately 20% of ARPKD cases [38]. T36M mutation was detected at a rate of 28% [17]. Obeidova et al. reported that it was the most common T36M mutation and was determined at a rate of 21% [18]. Another study conducted in Oman found the T36M mutation to be the most common [37]. In a study by Furu et al., this rate was determined to be 14.5% [15]. In this study, the rate of the T36M mutation was 1.2% (n = 1), and it was determined as compound heterozygous (p.Ser1156Leu/p.Thr36Met), as seen in Table 2.

The *PKHD1* DNA variants obtained as a result of genetic analyses in patients with a suspected or definitive diagnosis of ARPKD, as well as the literature on these variants, are outlined in Table 4. Relevant mutations responsible for this disease need to be identified, and their association with the disease confirmed [9]. Burgmaier et al. found that biallelic missense variants affecting amino acids 709–1837 were associated with a mild renal phenotype, while missense variants affecting amino acids 2625–4074 of fibrocystin were associated with a higher risk of significant hepatic complications [9]. The site of amino acid substitutions affects the severity of the phenotype occurring throughout the polyductin/fibrocystic protein. Some amino acid substitutions act as hypomorphic alleles with reduced function, while others cause complete loss of function, similar to chain termination [15]. Chain-terminating mutations may lead to a complete loss of function and inevitably result in prenatal death [15].

In their study, Jordan et al. screened a large gene panel of 100 fetuses (98 families) suffering from severe kidney defects and detected p.[Pro149Argfs*19] of the *PKHD1* gene; p.[Arg760His]; and mutations in p.[Ile833Thr], [Asp3808Metfs*12][Gly2951Val],p.[Glu218_ Tyr221delinsAsp]. Some of the rare variants of the *PKHD1* gene, which were detected in the genetic study conducted by Giacobbe et al., were also detected in this study, namely: c.4870C>T p.Arg1624Trp (R1624W) (likely pathogenic), c.3407A>G p.Tyr1136Cys (Y1136C) (benign) c.8606C>A p.Thr2869Lys (T2869K) (benign). In the study, two pathogenic variants (c.2702A>C and c.4870C>T) were associated with liver disease, kidney disease-associated pathogenic variant (c.5879C>G), and the complex allele of unknown clinical significance [c.3407A>G; c.8345G>C; c.8606C>A], which were found to be associated with the severe hepatic phenotype [20].

The c.3407A>G (p.Tyr1136Cys) heterozygous inheritance pattern that Eisenberger et al. determined in their study in 2015, and the compound heterozygous inheritance pattern (c.3407A>G and c.8606C>A) identified in another study, suggested the presence of Caroli syndrome [20]. As seen in the studies, representation of the different genotypes of this gene on the phenotype can be determined in different ways, and so the genotype-phenotype relationship related to this gene should not be ignored.

The genotype distributions (homozygous, heterozygous, compound heterozygous, complex genotype) of the mutations detected in Table 2 were evaluated. In this study, 39 (63.9%) individuals were heterozygous, six (9.8%) were homozygous, 12 (19.7%) were compound heterozygous, and four (6.6%) were complex genotypes (Table 2).

According to the 2020 study conducted by Alawi et al., 66% of patients were found to be homozygous and 28% to be compound heterozygous [37]. These ratios demonstrate the allelic heterogeneity of ARPKD disease.

Sanger, multiplex ligation-dependent probe amplification (MPLA) and denaturing high-performance liquid chromatography (DHPLC) are used in the literature to identify mutations of the PKHD1 gene [2,16,30]. Although it is possible to detect mutations, doing so is labor-intensive and time-consuming. As an alternative, the next generation sequencing (NGS) method is effective in the rapid screening for patients with suspected ARPKD, and both new and rare missense variants can be identified in this way [34,39]. The distributions of mutations detected using NGS in this study are as follows: 16 (19.5%) novel, 73 (89.02%) missense variants, six (7.32%) nonsense variants, two (%) 2.44) Frameshift deletion and one (1.22%) Nonframeshift deletion (Table 5).

The homozygous variant Arg723Cys (benign) in exon 22 of the PKDH1 gene has been associated with the typical features of ARPKD disease. This variant was reported in a case study related to this disease [40]. This study is essential in terms of demonstrating that the detected DNA variant, although benign, is a cause of disease. In our study, two different DNA variants, p.Arg723Leu (R723L) and p.Arg760Cys (R760C) (the most common appearing in this study), were detected in exon 22 of the PKDH1 gene and are benign according to ACMG classification.

Some mutations found in the Turkish population in previous studies are P1255Xfs, D3293V, T899P, L2772P, N3175S [15], Y1838C, I2427T, P356fs, G3359fs, S1156L, G2967W, F372L, I473S, H3124Y, I2851T (I199851T) [22].

## 5. Conclusions

In this study, the DNA variants of the *PKHD1* gene were determined in detail using the NGS method. The pathogenic distributions of these variants were determined by bioinformatics-based approaches and classified according to ACGM. It is therefore considered that this study contributes to the definitive diagnosis of ARPKD, determination of disease carriers, the planning of molecular prenatal diagnosis, and a better understanding of the molecular pathogenesis of the disease. It is also thought that the creation of disease-related exon mutation profiles for the study, and the use of bioinformatics approaches, will be of benefit in terms of reducing labor time and costs in the future molecular diagnosis of the disease. In addition, it is thought that creating appropriate platforms for gene-based diagnosis of ARPKD disease, and evaluating such platforms alongside prenatal tests, will help to establish definitive and differential clinical diagnosis. Furthermore, identifying relevant mutations associated with the disease will guide possible future pregnancies for parents who have a child diagnosed with ARPKD.

## Figures and Tables

**Figure:**
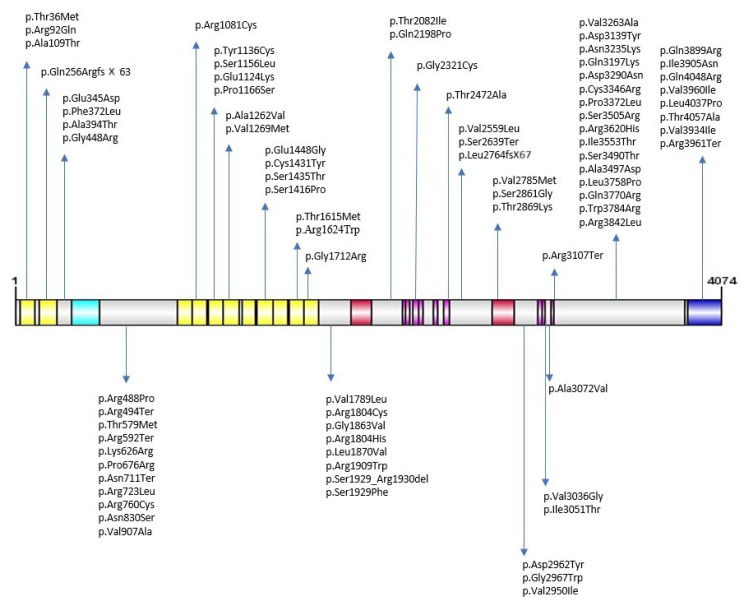
Representation of mutations detected on the structure of the *PKHD1* protein. Yellow: IPT/TIG domain; sky blue: PA14 domain; red: G8 1 domain; pink: PbH1 domain; blue: intracellular).

**Table 1:** Exon distributions and pathogenicity of *PKDH1* DNA variants.

Locus	Exon	Nucleotide change	Amino acid change	Mutation type	dbSNP	ACMG	Polyphen2 (Score)	SIFT (Score)
chr6:51944763	5	c.325G>A	p.Ala109Thr (A109T)	Missense	Rs143979330	Benign	Benign (0.156)	Tolerated (0.13)
chr6:51920485	19	c.1736C>T	p.Thr579Met (T579M)	Missense	Rs45500692	Benign	Probably damaging (1.000)	Affect protein function (0.00)
chr6:51918923	20	c.1877A>G	p.Lys626Arg (K626R)	Missense	Rs117122807	Benign	Benign (0.001)	Affect protein function (0.00)
chr6:51917987	21	c.2027C>G	p.Pro676Arg (P676R)	Missense	Rs115045643	Benign	Benign (0.002)	Affect protein function (0.00)
chr6:51910905	24	c.2489A>G	p.Asn830Ser (N830S)	Missense	Rs62406032	Benign	Benign (0.024)	Affect protein function (0.00)
chr6:51893107	30	c.3407A>G	p.Tyr1136Cys (Y1136C)	Missense	Rs41273726	Benign	Probably damaging (0.997)	Affect protein function (0.00)
chr6:51890823	32	c.3785C>T	p.Ala1262Val (A1262V)	Missense	Rs9296669	Benign	Benign (0.000)	Tolerated (0.15)
chr6:51890265	32	c.4343A>G	p.Glu1448Gly (E1448G)	Missense	Rs116809571	Benign	Benign (0.000)	Affect protein function (0.00)
chr6:51875250	35	c.5608T>G	p.Leu1870Val (L1870V)	Missense	Rs2435322	Benign	Benign (0.000)	Tolerated (0.09)
chr6:51875133	35	c.5725C>T	p.Arg1909Trp (R1909W)	Missense	Rs115338476	Benign	Benign (0.017)	Affect protein function (0.00)
chr6:51777251	38	c.6245C>T	p.Thr2082Ile (T2082I)	Missense	Rs147487242	Benign	Benign (0.001)	Affect protein function (0.02)
chr6:51637561	55	c.8581A>G	p.Ser2861Gly (S2861G)	Missense	Rs150925674	Benign	Benign (0.011)	Tolerated (0.82)
chr6:51637536	55	c.8606C>A	p.Thr2869Lys (T2869K)	Missense	Rs142522748	Benign	Probably damaging (0.982)	Affect protein function (0.01)
chr6:51612626	58	c.9788T>C	p.Val3263Ala (V3263A)	Missense	Rs146519878	Benign	Possibly damaging (0.905)	Affect protein function (0.01)
chr6:51524409	61	c.10515C>A	p.Ser3505Arg (S3505R)	Missense	Rs139014478	Benign	Benign (0.006)	Tolerated (0.93)
chr6:51497503	65	c.11525G>T	p.Arg3842Leu (R3842L)	Missense	Rs76572975	Benign	Possibly damaging (0.992)	Affect protein function (0.00)
chr6:51491884	66	c.11696A>G	p.Gln3899Arg (Q3899R)	Missense	Rs4715227	Benign	Benign (0.295)	Affect protein function (0.00)
chr6:51491866	66	c.11714T>A	p.Ile3905Asn (I3905N)	Missense	Rs2661488	Benign	Benign (0.000)	Affect protein function (0.00)
chr6:51483961	67	c.12143A>G	p.Gln4048Arg (Q4048R)	Missense	Rs9381994	Benign	Benign (0.031)	Affect protein function (0.00)
chr6:51484226	67	c.11878G>A	p.Val3960Ile (V3960I)	Missense	Rs34548196	Benign	Benign (0.000)	Affect protein function (0.00)
chr6:51915066	22	c.2168G>T	p.Arg723Leu (R723L)	Missense	Rs150597050	Benign	Benign (0.166)	Affect protein function (0.00)
chr6:51889764	32	c.4844C>T	p.Thr1615Met (T1615M)	Missense	Rs147529495	Benign	Benign (0.045)	Tolerated (0.12)
chr6:51613199	58	c.9215C>T	p.Ala3072Val (A3072V)	Missense	Rs139306706	Benign	Possibly damaging (0.632)	Affect protein function (0.03)
chr6:51612999	58	c.9415G>T	p.Asp3139Tyr (D3139Y)	Missense	Rs45503297	Benign	Benign (0.007)	Tolerated (0.07)
chr6:51914956	22	c.2278C>T	p.Arg760Cys (R760C)	Missense	Rs9370096	Benign	Benign (0.007)	Affect protein function (0.00)
chr6:51484304	67	c.11800G>A	p.Val3934Ile (V3934I)	Missense	Rs149111536	Benign	Benign (0.002)	Affect protein function (0.00)
chr6:51947196	4	c.275G>A	p.Arg92Gln (R92Q)	Missense	Rs145886657	Likely benign	Benign (0.031)	Tolerated (0.37)
chr6:51897951	29	c.3241C>T	p.Arg1081Cys (R1081C)	Missense	Rs200986136	Likely benign	Benign (0.267)	Affect protein function (0.00)
chr6:51524065	61	c.10859G>A	p.Arg3620His (R3620H)	Missense	Rs149163661	VUS	Benign (0.179)	Affect protein function (0.00)
chr6:51927400	14	c.1035A>T	p.Glu345Asp (E345D)	Missense	**Novel**	VUS	Benign (0.335)	Affect protein function (0.00)
chr6:51923170	16	c.1463G>C	p.Arg488Pro (R488P)	Missense	Rs186202437	VUS	Possibly damaging (0.800)	Tolerated (0.07)
chr6:51923291	16	c.1342G>C	p.Gly448Arg (G448R)	Missense	Rs149781976	VUS	Probably damaging (0.999)	Tolerated (0.24)
chr6:51908524	26	c.2720T>C	p.Val907Ala (V907A)	Missense	Rs1320546830	VUS	Benign (0.002)	Tolerated (0.57)
chr6:51893144	30	c.3370G>A	p.Glu1124Lys (E1124K)	Missense	**Novel**	VUS	Benign (0.172)	Affect protein function (0.00)
chr6:51893018	30	c.3496C>T	p.Pro1166Ser (P1166S)	Missense	Rs895296852	VUS	Benign (0.013)	Tolerated (0.23)
chr6:51889474	32	c.5134G>A	p.Gly1712Arg (G1712R)	Missense	Rs141103838	VUS	Probably damaging (1.000)	Affect protein function (0.00)
chr6:51890304	32	c.4304G>C	p.Ser1435Thr (S1435T)	Missense	Rs138242579	VUS	Probably damaging (0.989)	Affect protein function (0.00)
chr6:51890803	32	c.3805G>A	p.Val1269Met (V269M)	Missense	Rs139820610	VUS	Probably damaging (0.964)	Affect protein function (0.00)
chr6:51890362	32	c.4246T>C	p.Ser1416Pro (S1416P)	Missense	**Novel**	VUS	Possibly Damaging (0.543)	Tolerated (0.19)
chr6:51887614	33	c.5365G>C	p.Val1789Leu (V1789L)	Missense	Rs1288521396	VUS	Possibly damaging (0.944)	Tolerated (0.05)
chr6:51882398	34	c.5410C>T	p.Arg1804Cys (R1804C)	Missense	Rs201906247	VUS	Benign (0.279)	Tolerated (0.10)
chr6:51882220	34	c.5588G>T	p.Gly1863Val (G1863V)	Missense	rs NA	VUS	Probably damaging (1.000)	Affect protein function (0.00)
chr6:51882397	34	c.5411G>A	p.Arg1804His (R1804H)	Missense	Rs151160618	VUS	Benign (0.000)	Tolerated (0.10)
chr6:51824785	36	c.5785_5790delTCCAGG	p.Ser1929_Arg1930del	Nonframeshift	**Novel**	VUS	-	-
chr6:51824790	36	c.5786C>T	p.Ser1929Phe (S1929F)	Missense	**Novel**	VUS	Probably damaging (0.998)	Affect protein function (0.00)
chr6:51774170	40	c.6593A>C	p.Gln2198Pro (Q2198P)	Missense	Rs1250730619	VUS	Benign (0.412)	Tolerated (0.22)
chr6:51768430	43	c.6961G>T	p.Gly2321Cys (G2321C)	Missense	**Novel**	VUS	Probably damaging (1.000)	Tolerated (0.07)
chr6:51735374	47	c.7414A>G	p.Thr2472Ala (T2472A)	Missense	Rs137972951	VUS	Probably damaging (0.999)	Tolerated (0.20)
chr6:51732719	48	c.7675G>C	p.Val2559Leu (V2559L)	Missense	Rs150046042	VUS	Benign (0.000)	Tolerated (0.24)
chr6:51656121	53	c.8353G>A	p.Val2785Met (V2785M)	Missense	**Novel**	VUS	Probably damaging (0.999)	Affect protein function (0.01)
chr6:51618065	57	c.8884G>T	p.Asp2962Tyr (D2962Y)	Missense	**Novel**	VUS	Probably damaging (1.000)	Affect protein function (0.00)
chr6:51618050	57	c.8899G>T	p.Gly2967Trp (G2967W)	Missense	**Novel**	VUS	Probably damaging (1.000)	Affect protein function (0.00)
chr6:51618101	57	c.8848G>A	p.Val2950Ile (V2950I)	Missense	Rs768138709	VUS	Probably damaging (1.000)	Tolerated (0.07)
chr6:51612709	58	c.9705T>A	p.Asn3235Lys (N3235K)	Missense	Rs759568939	VUS	Probably damaging (0.956)	Tolerated (0.13)
chr6:51612825	58	c.9589C>A	p.Gln3197Lys (Q3197K)	Missense	**Novel**	VUS	Possibly damaging (0.498)	Tolerated (0.41)
chr6:51613262	58	c.9152T>C	p.Ile3051Thr (I3051T)	Missense	rs1374086784	VUS	Probably damaging (1.000)	Affect protein function (0.01)
chr6:51611649	59	c.9868G>A	p.Asp3290Asn (D3290N)	Missense	Rs370659581	VUS	Benign (0.032)	Tolerated (0.73)
chr6:51609224	60	c.10115C>T	p.Pro3372Leu (P3372L)	Missense	Rs779738981	VUS	Probably damaging (1.000)	Affect protein function (0.00)
chr6:51524455	61	c.10469G>C	p.Ser3490Thr (S3490T)	Missense	Rs149486694	VUS	Benign (0.156)	Tolerated (0.74)
chr6:51524434	61	c.10490C>A	p.Ala3497Asp (A3497D)	Missense	**Novel**	VUS	Probably damaging (0.995)	Affect protein function (0.02)
chr6:51513920	62	c.11273T>C	p.Leu3758Pro (L3758P)	Missense	**Novel**	VUS	Probably damaging (1.000)	Affect protein function (0.00)
chr6:51512877	63	c.11350T>C	p.Trp3784Arg (W3784R)	Missense	Rs1248281525	VUS	Probably damaging (0.986)	Affect protein function (0.00)
chr6:51483994	67	c.12110T>C	p.Leu4037Pro (L4037P)	Missense	Rs199900211	VUS	Possibly damaging (0.838)	Affect protein function (0.00)
chr6:51483935	67	c.12169A>G	p.Thr4057Ala (T4057A)	Missense	**Novel**	VUS	Benign (0.005)	Affect protein function (0.00)
chr6:51924779	15	c.1180G>A	p.Ala394Thr (A394T)	Missense	Rs1582024305	Likely Pathogenic	Probably damaging (1.000)	Affect protein function (0.03)
chr6:51893047	30	c.3467C>T	p.Ser1156Leu (S1156L)	Missense	Rs367707903	Likely Pathogenic	Benign (0.000)	Affect protein function (0.01)
chr6:51889738	32	c.4870C>T	p.Arg1624Trp (R1624W)	Missense	Rs200391019	Likely Pathogenic	Probably damaging (0.992)	Affect protein function (0.05)
chr6:51613307	58	c.9107T>G	p.Val3036Gly (V3036G)	Missense	Rs893497345	Likely Pathogenic	Possibly damaging (0.952)	Affect protein function (0.00)
chr6:51609303	60	c.10036T>C	p.Cys3346Arg (C3346R)	Missense	Rs149798764	Likely Pathogenic	Probably damaging (0.999)	Affect protein function (0.00)
chr6:51524266	61	c.10658T>C	p.Ile3553Thr (I3553T)	Missense	Rs137852948	Likely Pathogenic	Possibly damaging (0.501)	Affect protein function (0.01)
chr6:51927319	14	c.1116C>G	p.Phe372Leu (F372L)	Missense	Rs1582038191	Likely Pathogenic	Probably damaging (0.996)	Affect protein function (0.00)
chr6:51917883	21	c.2130_2131insTA	p.Asn711Ter (N711X)	Nonsense	**Novel**	Likely Pathogenic	-	-
chr6:51513884	62	c.11309A>G	p.Gln3770Arg (Q3770R)	Missense	rs1770520773	Likely Pathogenic	Benign (0.278)	Affect protein function (0.00)
chr6:51890316	32	c.4292G>A	p.Cys1431Tyr (C1431Y)	Missense	Rs753307105	Pathogenic	Probably damaging (0.993)	Affect protein function (0.00)
chr6:51484223	67	c.11881C>T	p.Arg3961Ter (R3961X)	Nonsense	Rs144193508	Pathogenic	-	-
chr6:51947999	3	c.107C>T	p.Thr36Met (T36M)	Missense	Rs137852944	Pathogenic	Probably damaging (1.000)	Affect protein function (0.00)
chr6:51934266	11	c.766delC	p.Gln256ArgfsX63	Frameshift	**Novel**	Pathogenic	-	-
chr6:51923153	16	c.1480C>T	p.Arg494Ter (R494X)	Nonsense	Rs754392766	Pathogenic	-	-
chr6:51920447	19	c.1774C>T	p.Arg592Ter (R592X)	Nonsense	Rs779050294	Pathogenic	-	-
chr6:51712764	50	c.7916C>A	p.Ser2639Ter (S2639X)	Nonsense	Rs181208607	Pathogenic	-	-
chr6:51695668	52	c.8291_8292delTC	p.Leu2764fsX67	Frameshift	**Novel**	Pathogenic	-	-
chr6:51613095	58	c.9319C>T	p.Arg3107Ter (R3107X)	Nonsense	Rs786204688	Pathogenic	-	-

**Table 2:** Genotype distributions of DNA variants of the *PKHD1* gene.

Heterozygous	N	%
p.Arg3107Ter	7	11.5
p.Gly1712Arg	4	6.6
p.Asn3235Lys	3	4.9
p.Ala394Thr	3	4.9
p.Ser1156Leu	2	3.3
p.Asn711Ter	1	1.6
p.Leu4037Pro	1	1.6
p.Val2950Ile	1	1.6
p.Arg3961Ter	1	1.6
p.Thr2472Ala	1	1.6
p.Asp2962Tyr	1	1.6
p.Cys3346Arg	1	1.6
p.Thr4057Ala	1	1.6
p.Ser3490Thr	1	1.6
p.Gln2198Pro	1	1.6
p.Gln3197Lys	1	1.6
p.Glu345Asp	1	1.6
p.Ser1435Thr	1	1.6
p.Val1269Met	1	1.6
p.Val907Ala	1	1.6
p.Ser1929Phe	1	1.6
p.Pro1166Ser	1	1.6
p.Ser1416Pro	1	1.6
p.Arg1804His	1	1.6
p.Pro3372Leu	1	1.6
**Total heterozygous**	**39**	**63.9**
**Homozygous**	N	%
p.Cys1431Tyr	1	1.6
p.Gln3770Arg	1	1.6
p.Ser1929_Arg1930del	1	1.6
p.Val1789Leu	1	1.6
p.Glu1124Lys	1	1.6
p.Ser2639Ter	1	1.6
**Total homozygous**	**6**	**9.8**
**Compound heterozygous**	**N**	**%**
p.Leu3758Pro/p.Arg1624Trp	1	1.6
p.Arg3961Ter/p.Arg592Ter	1	1.6
p.Ser1156Leu/p.Thr36Met	1	1.6
p.Arg3107Ter/p.Asp2962Tyr	1	1.6
p.Val2785Met/p.Leu2764fsX67	1	1.6
p.Arg3620His/p.Gly2321Cys	1	1.6
p.Val3036Gly/p.Arg494Ter	1	1.6
p.Ile3553Thr/p.Ile3051Thr	1	1.6
p.Arg1804Cys/p.Gly448Arg	1	1.6
p.Trp3784Arg/p.Ser1156Leu	1	1.6
p.Ala3497Asp/p.Gly1863Val	1	1.6
p.Asn3235Lys/p.Gln256ArgfsX63	1	1.6
**Total compound heterozygous**	**12**	**19.7**
**Complex genotype**	**N**	**%**
p.Arg488Pro/p.Phe372Leu(**Homozygous**/**homozygous**)	2	3.3
p.Asp3290Asn/p.Gly2967Trp/p.Val2559Leu/p.Ser1156Leu(**heterozygous**/**heterozygous**/**heterozygous**/**heterozygous**)	1	1.6
p.Ser1156Leu/p.Arg488Pro/p.Phe372Leu(**heterozygous**/**heterozygous**/**heterozygous**)	1	1.6
**Total complex genotype**	**4**	**6.6**
**Total**	**61**	**100**

**Table 3:** Exon distributions of *PKDH1* DNA variants in this study.

Exon numbers	Number of DNA variants
32	9
58	8
67	6
61	5
30	4
16, 34, and 57	3
14, 21, 22, 19, 35, 36, 55, 60, 62, and 66	2
3, 4, 5, 11, 15, 20, 24, 26, 29, 33, 38, 40, 43, 47, 48, 50, 52, 53, 59, 63, and 65	1
**Total:** 39 exons	**Total:** 82

**Table 4:** *PKDH1* DNA variants detected in this study and studies involving these variants.

Exon	Amino acid change	References
3	p.Thr36Met	[17,25,26,28,30], this study
4	p.Arg92Gln	[25], this study
14	p.Phe372Leu	[16], this study
15	p.Ala394Thr	[23], this study
16	p.Arg488Pro	[16,22], this study
16	p.Gly448Arg	[23], this study
16	p.Arg494Ter	[32], this study
19	p.Thr579Met	[16,22,23], this study
22	p.Arg760Cys	[16,23,27], this study
22	p.Arg723Leu	[29], this study
24	p.Asn830Ser	[16,22,23], this study
30	p.Tyr1136Cys	[16,20,22,23,25,26], this study
30	p.Ser1156Leu	[25,30,34], this study
32	p.Ala1262Val	[16,22,23,27], this study
32	p.Glu1448Gly	[30], this study
32	p.Gly1712Arg	[26], this study
32	p.Arg1624Trp	[17,20,26,30,33], this study
32	p.Cys1431Tyr	[28,34], this study
33	p.Val1789Leu	[15], this study
34	p.Arg1804Cys	[23], this study
35	p.Leu1870Val	[23,27], this study
35	p.Arg1909Trp	[23], this study
48	p.Val2559Leu	[23], this study
50	p.Ser2639Ter	[17,30], this study
55	p.Ser2861Gly	[15,23,25,26,30], this study
55	p.Thr2869Lys	[16,20,21,22], this study
58	p.Asp3139Tyr	[16,22,23,26], this study
58	p.Ala3072Val	[16,22,23], this study
58	p.Val3036Gly	[15,23,30,34], this study
58	p.Val3263Ala	[23], this study
58	p.Ile3051Thr	[30], this study
58	p.Arg3107Ter	[30,34,36], this study
60	p.Cys3346Arg	[23], this study
61	p.Ser3505Arg	[16,22,23], this study
61	p.Ile3553Thr	[17], this study
65	p.Arg3842Leu	[16,22,23,26], this study
66	p.Gln3899Arg	[22,23,27], this study
66	p.Ile3905Asn	[16,23], this study
67	p.Gln4048Arg	[16,22,23,27], this study
67	p.Val3960Ile	[16,22], this study
67	p.Arg3961Ter	[34], this study
58	p.Asn3235Lys	[31], this study

**Table 5:** Novel *PKDH1* DNA variants in this study.

Locus	Exon	Nucleotide change	Amino acid change	Mutation type	dbSNP
chr6:51934266	11	c.766delC	p.Gln256ArgfsX63	Frameshift	Novel
chr6:51927400	14	c.1035A>T	p.Glu345Asp (E345D)	Missense	Novel
chr6:51917883	21	c.2130_2131insTA	p.Asn711Ter (N711X)	Nonsense	Novel
chr6:51893144	30	c.3370G>A	p.Glu1124Lys (E1124K)	Missense	Novel
chr6:51890362	32	c.4246T>C	p.Ser1416Pro (S1416P)	Missense	Novel
chr6:51824790	36	c.5786C>T	p.Ser1929Phe (S1929F)	Missense	Novel
chr6:51824785	36	c.5785_5790delTCCAGG	p.Ser1929_Arg1930del	Nonframeshift	Novel
chr6:51768430	43	c.6961G>T	p.Gly2321Cys (G2321C)	Missense	Novel
chr6:51695668	52	c.8291_8292delTC	p.Leu2764fsX67	Frameshift	Novel
chr6:51656121	53	c.8353G>A	p.Val2785Met (V2785M)	Missense	Novel
chr6:51618065	57	c.8884G>T	p.Asp2962Tyr (D2962Y)	Missense	Novel
chr6:51618050	57	c.8899G>T	p.Gly2967Trp (G2967W)	Missense	Novel
chr6:51612825	58	c.9589C>A	p.Gln3197Lys (Q3197K)	Missense	Novel
chr6:51524434	61	c.10490C>A	p.Ala3497Asp (A3497D)	Missense	Novel
chr6:51513920	62	c.11273T>C	p.Leu3758Pro (L3758P)	Missense	Novel
chr6:51483935	67	c.12169A>G	p.Thr4057Ala (T4057A)	Missense	Novel
